# Fingering instability at the interface between growing and not-growing cell populations with different mobilities

**DOI:** 10.1007/s00285-026-02463-6

**Published:** 2026-10-21

**Authors:** P. Ciarletta, M. Magri, D. Shkurti, C. de Falco

**Affiliations:** 1 MOX, Department of Mathematics, Politecnico di Milano, Piazza Leonardo da Vinci 32, 20133 Milan, Italy; 2 Laboratoire de Probabilités, Statistique et Modélisation (LPSM), Sorbonne Université, CNRS UMR 8001, Paris, France

**Keywords:** 35B35, 35C07, 35K65, 92C17

## Abstract

Invasion of solid tumours is often mediated by heterogeneous cell populations that differ in motility, adhesion, and proliferation. Motivated by these mechanisms, we study a two-population continuum model in which the total density generates a pressure field and cell transport follows a Darcy-type law, coupled to growth and nonlinear diffusion. The model admits planar travelling-wave solutions describing an advancing invasion front. We derive, in the planar travelling-wave geometry and in the incompressible limit, an explicit dispersion relation for transverse perturbations of segregated fronts. The instability results in the amplification of interfacial modes and the emergence of fingering patterns, and numerical simulations near the incompressible limit recover the growth rates predicted by the linear analysis. To validate these predictions, we design a fully implicit positivity–preserving numerical scheme based on a projected Newton–Krylov solver, which enforces non-negativity of the densities at the algebraic level and remains robust in stiff regimes with sharp gradients, to avoid spurious front breakup. Numerical experiments reproduce the predicted early-time amplification at the most unstable modes considered. Altogether, the results link phenotypic heterogeneity and mechanically mediated transport to morphological instabilities of tumour invasion fronts, with potential relevance to interfaces established by adhesion-mediated cell sorting.

## Introduction

Mathematical modeling of tumour growth provides a rigorous framework to investigate the spatiotemporal dynamics of cell populations, elucidating how local proliferation, mechanical interactions, and tissue heterogeneity give rise to complex behaviors. Early continuum models, such as those developed by Greenspan (1972), focused on the diffusive transport of nutrients in spherical tumours, laying the groundwork for pressure-based formulations. Subsequent studies expanded this framework by including mechanical feedback, volume constraints, and multiphase approaches (Friedman 2004; Murray 2003; Byrne et al. 2006), progressively bridging the gap between theory and biological realism.

Among these developments, increasing attention has been paid to models that describe the interaction between multiple cell types, particularly in the context of solid tumours. In such systems, proliferating cells may invade surrounding healthy tissue or necrotic cores, leading to interfacial dynamics governed by growth and mobility differences. These processes can be captured by Darcy-type laws for cell motion driven by pressure gradients, coupled with nonlinear source terms that model density-dependent proliferation (Ranft et al. 2010; Perthame et al. 2014). A seminal contribution in this direction is due to Lorenzi et al. (2017), who introduced a two-population model describing segregated fronts in terms of distinct cell mobilities and proliferation, revealing the emergence of travelling wave solutions under pressure constraints.

From a mathematical viewpoint, the governing equations belong to the class of degenerate reaction–cross-diffusion systems in which interacting populations move under a pressure generated by their total density. Recent studies have developed structure-preserving variational and finite-volume schemes for such systems, as well as weak-solution theories and incompressible-limit results for related pressure-driven multi-species models (Carrillo et al. 2018; Chertock et al. 2019; Bubba et al. 2020; Degond et al. 2020; Dębiec and Schmidtchen 2020; Jacobs 2023; David 2023). These contributions provide the broader analytical and numerical context for the density formulation considered here.

Several other contributions have enriched the mathematical analysis of interacting populations by incorporating additional mechanisms such as cell adhesion, extracellular matrix degradation, and anisotropic diffusion. Anderson’s hybrid framework (Anderson 2005) emphasized the importance of adhesion in shaping tumour invasion patterns, while (Chaplain and Lolas 2005) integrated proteolytic activity into models of invasive spread. More recently, models based on free boundary problems and quasi-incompressible flow have been proposed to account for mechanical regulation in tightly packed tissues, leading to interface conditions that involve pressure continuity and flux conservation (Hillairet and Vauchelet 2020). Morphological instability and symmetry breaking have also been investigated in free-boundary and mixture models of tumour growth, including branching morphologies, symmetry-breaking bifurcations and nutrient-driven boundary instabilities (Cristini et al. 2003, 2009; Friedman and Reitich 2001; Feng et al. 2023).

Adhesion-mediated cell sorting can be interpreted mechanically in terms of effective tissue contact and interfacial tensions. These tensions emerge from the combined action of cell–cell adhesion and cortical contractility and can drive the segregation and envelopment of distinct cell populations (Brodland and Chen 2000; Manning et al. 2010). This mechanism provides a possible origin for the segregated configuration considered here. The present model does not describe the sorting process itself, but investigates the subsequent pressure-driven stability of the interface when the two populations have different mobilities.

Travelling fronts are not restricted to pathological growth: propagating waves of cell differentiation have also been observed during tissue morphogenesis, notably in skull-bone expansion (Dang et al. 2025).

The interface dynamics of related incompressible two-population models has been studied previously in the sharp-interface setting. In particular, Kim and Tong (2021) analysed the free-boundary formulation with two moving interfaces separating the tumour phase, the surrounding tissue and the exterior region, and proved local well-posedness for configurations close to radial symmetry. Their analysis already contains the mobility-dependent parabolicity mechanism associated with protrusion growth. This provides an important sharp-interface reference point for the present study. At the level of the limiting free-boundary evolution, their result identifies the mobility-dependent parabolicity of the internal interface.

When proliferation and mobility gradients drive the evolution of a sharp interface between two cell populations, analogies naturally arise with classical fluid mechanical problems. In particular, the Saffman–Taylor instability—originally describing viscous fingering at the interface of two immiscible fluids in a Hele–Shaw cell (Saffman and Taylor 1958)—has inspired new perspectives in tissue dynamics, especially in models where a less mobile population is displaced by a more motile one. Although such instabilities are well-studied in phase-field and fluid systems (Krishnan et al. 2020), their implications in biological settings remain underexplored, particularly in growth-driven contexts where mechanical and proliferative processes are intertwined (Recho et al. 2016a). Fingering-like instabilities of travelling-wave solutions were previously observed numerically for this model in Ref. Lorenzi et al. (2017), although their onset and wavelength selection were not analytically characterized. Transverse instabilities of planar travelling waves have also been analysed in simpler two-component reaction–diffusion systems, where the normal-mode calculation yields an explicit range of unstable wavenumbers (Kowalczyk et al. 2015).

Their limited investigation may partly reflect the difficulty of resolving the sharp, pressure-driven interfaces of the governing equations without introducing numerical artefacts.

From a computational perspective, modeling the evolution and stability of such interfaces requires numerical methods capable of resolving steep gradients and preserving structural features of the governing equations. Krylov subspace solvers and Jacobian-free Newton–Krylov methods have emerged as powerful tools in this setting, offering efficient and robust schemes for nonlinear systems involving free boundaries and saturation constraints (Pethiyagoda et al. 2015; Loiseau et al. 2019). These methods, when combined with finite difference or finite element schemes, provide a computationally efficient framework to simulate interfacial dynamics in tumour modeling and beyond.

Segregated travelling waves in one space dimension are known to exist for general positive mobilities, with parameter-dependent speed and profile (Lorenzi et al. 2017). Their dynamical selection in the one-dimensional problem depends on the mobility contrast, but no rigorous spectral or nonlinear stability theorem is available in that setting. The question addressed here is therefore different: we take the planar travelling front as the reference state and analyse whether it remains stable when transverse perturbations are allowed in two space dimensions.

The aim of the present paper is complementary to the existing sharp-interface theory. We do not address the general free-boundary evolution near radial configurations. Instead, we focus on the segregated travelling wave arising from the density formulation in the incompressible limit and study its transverse stability. This planar setting allows one to obtain an explicit dispersion relation and to compare the predicted unstable modes directly with numerical simulations of the finite-compressibility system. The paper is structured as follows: Sect. 2 presents the mathematical model and its one-dimensional travelling-wave solution. Section 3 derives the transverse dispersion relation. Section 4 describes the numerical method and its validation. Conclusions and perspectives are discussed in Sect. 5.

## Mathematical model

We consider the modeling framework proposed by Lorenzi et al. (2017) for the description of cancer invasion, based on the porous medium equation. In the following, we first introduce the partial differential system, and later derive its travelling wave solution.

### Governing equations

The partial differential system describes the dynamics of two populations of cells in the following form:

1a $$\begin{aligned}&\frac{\partial m}{\partial t} - \mu \, \text {div} \left[ m \nabla {p} \right] = G (p) \, m \, , \end{aligned}$$

1b $$\begin{aligned}&\frac{\partial n}{\partial t} - \nu \, \text {div} \left[ n \nabla {p} \right] = 0 \, . \end{aligned}$$

Symbols *m* and *n* refer to the density of dividing and non-dividing cells, respectively. The former species are regarded as cancer cells, while the second species identify healthy cells. The two governing equations are coupled through the pressure *p*, which is constitutively defined as

2 $$\begin{aligned} p = K_\gamma (n+m)^\gamma \, , \qquad \text {with} \qquad K_\gamma = \frac{\gamma + 1}{\gamma } \, , \end{aligned}$$

where $K_\gamma$ is a volumetric stiffness that is controlled by the model parameter $\gamma \ge 1$. In particular, the condition $\gamma \rightarrow \infty$ identifies the so-called *Hele-Shaw* or *incompressible* limit (Perthame et al. 2014). The model describes cell motion at the tissue scale, with pressure emerging from local crowding and preventing further compression in the incompressible regime. The two populations interact mechanically through this common pressure field, while the mobilities μ and ν measure how readily each population can rearrange against its surroundings. The extracellular matrix, substrate and interstitial fluid are not represented as separate phases; their mechanical effects are absorbed into these effective mobilities and into the pressure law. The model contains a single pressure field generated by the total cell density and does not include an interfacial energy or a singular capillary stress at the boundary between the two populations. Accordingly, in the sharp-interface limit we impose continuity of *p* across the internal interface. A Young-Laplace condition would instead require an explicit interfacial tension, which is not part of the governing system considered here.

The right-hand side of Eq. (1a) is a source term that models the division rate of tumour cells by means of a pressure-dependent net growth rate *G*(*p*). Since the cancer growth rate usually decreases as the available space is filled with cells, *G*(*p*) shall satisfy the restrictions

3 $$\begin{aligned} \frac{\text {d} G}{\text {d} p} < 0 \qquad \text {and} \qquad G(P_M) = 0 \, , \end{aligned}$$

where parameter $P_M \ge 0$ is the so-called *homeostatic pressure*, a limit value of the pressure above which cell-division is suppressed because of saturation of the available space (Ranft et al. 2010).

### One-dimensional travelling wave solution

The existence of segregated one-dimensional travelling waves for the present two-population model was established in Theorem 3.1 of Lorenzi et al. (2017) for general positive mobilities. Related rigorous results for scalar porous-medium models of tissue growth include the incompressible-limit asymptotics and nonlinear stability of monotone travelling waves established in Dalibard et al. (2024). Here, we investigate the transverse linear stability of the segregated planar front and its possible evolution into finger-like solutions in the fully nonlinear regime.

In this section, we formally derive the solution as discontinuous travelling waves assuming the simplest form for *G*(*p*) fulfilling (3), that is:

4 $$\begin{aligned} G(p)= (P_M - p) \end{aligned}$$

Accordingly, we take the following ansatz for m and n:

5 $$\begin{aligned} m (x,t) = m ( x - \sigma t) \qquad \text {and} \qquad n (x,t) = n ( x - \sigma t) \, , \end{aligned}$$

where σ denotes the front velocity while *x* refers to the spatial coordinate. As customary, the moving frame $\xi = x -\sigma t$ can be introduced to ease the notation. We further assume that *m* and *n* have strictly separated supports, and that the one for *n* has finite size *r*. Specifically, we assume that:

$$ \text {Supp}(m) = (-\infty , 0 ] \qquad \text {and} \qquad \text {Supp}(n) = [0, r] \,, $$

with r>0. We also observe that $p'(\pm \infty )=0$ and $n ( \pm \infty ) = 0$ in the considered scenario, with prime indicating the total derivative with respect to ξ.

Substituting (5) into (1), we get

6a $$\begin{aligned}&- \sigma m' - \mu \, \left( m \, p' \right) ' = (P_M - p) \, m \, , \end{aligned}$$

6b $$\begin{aligned}&- \sigma n' - \nu \, \left( n \, p' \right) ' = 0 \, . \end{aligned}$$

We now resolve these equations separately in the regions $\text {Supp}(m)$ and $\text {Supp}(n)$. First, by integration of equation (6b) we obtain

$$ \left( \sigma + \nu \, p' \right) = 0 \,, $$

whose solution is

7 $$\begin{aligned} p (\xi ) = \frac{\sigma }{\nu } \left( r - \xi \right) \qquad \text {for} \qquad \xi \in [0,r] \, . \end{aligned}$$

by imposing the condition n(r)=0. Moreover, since *n* lies on a compact support, we can compute its total mass *M* as

$$ M = \int _0^r n (\xi ) \, \text {d}\xi = \int _0^r \left( \frac{p(\xi )}{K_\gamma } \right) ^{\frac{1}{\gamma }} \, \text {d}\xi = \left( \frac{\sigma }{\nu K_\gamma } \right) ^{\frac{1}{\gamma }} \frac{\gamma }{1+\gamma }{r^{\frac{1+\gamma }{\gamma }} }\,, $$

thus establishing a direct relationship between *M* and *r*.

Second, we consider the incompressible limit $\gamma =1/\,\varepsilon$ with $\,\varepsilon \rightarrow 0$ to solve (6a) for *p*.

Since $m=(p/K_\gamma )^{\varepsilon }$ in $\operatorname {Supp}(m)$, the travelling-wave equation is equivalently

$$ -\mu p''-(P_M-p) = \,\varepsilon \left( \sigma \frac{p'}{p} +\mu \frac{(p')^2}{p} \right) . $$

Hence, as $$\,\varepsilon \rightarrow 0$$,

$$ -\mu p''=P_M-p. $$

Moreover, the expansion $m=1+\,\varepsilon \log (p/K_\gamma )+O(\,\varepsilon ^2)$ is regular in the proliferating region because the limiting profile satisfies $p\ge p(0)=\sigma r/\nu >0$. The general solution of the limiting equation takes the form

$$ p (\xi ) = P_M + c_1 \text {e}^{\frac{\xi }{\sqrt{\mu }}} + c_2 \text {e}^{-\frac{\xi }{\sqrt{\mu }}} \,, $$

with $$c_1$$ and $$c_2$$ denoting the integration constants. These can be calculated by applying the conditions $p^{\prime }\left(-\infty \right)=0$ and $$p(0)=\sigma r /\nu $$, the latter arising because of the continuity of *p* at ξ=0. Thus, we obtain

8 $$\begin{aligned} p (\xi ) = P_M + \left( \frac{\sigma \, r}{\nu } - P_M \right) \, \text {e}^{\frac{\xi }{\sqrt{\mu }}} \qquad \text {for} \qquad \xi \in (-\infty ,0] \, . \end{aligned}$$

We can also determine the expression of the wave speed σ by matching the jump condition of $p^{\prime }$ at the interface ξ=0. Specifically, we sum (6a) and (6b) and integrate between ξ and *r*, obtaining

$$ \left. \sigma \left( m + n \right) \right| _{\xi } + \left. \left( \mu m + \nu n \right) \, p' \right| _{\xi } = \int _\xi ^r (P_M - p) \, m \, \text {d} \xi \,, $$

so that we get

$$ \left. \left( \mu m + \nu n \right) \, p' \right| _{\xi \rightarrow 0^-} = \left. \left( \mu m + \nu n \right) \, p' \right| _{\xi \rightarrow 0^+} \,, $$

because of the continuity of *p* and, therefore, of (m+n). This also implies that $$m(0^-)=n(0^+)$$ and, accordingly, the jump condition for $p^{\prime }$ at ξ=0 reads

9 $$\begin{aligned} p'(0^-) = \frac{\nu }{\mu } p'(0^+) = - \frac{\sigma }{\mu } \, . \end{aligned}$$

The wave velocity σ can be derived by matching (9) in (8), obtaining:

10 $$\begin{aligned} \sigma = \frac{P_M \, \nu \, \sqrt{\mu }}{r \sqrt{\mu } + \nu } \, . \end{aligned}$$

**Fig. 1:**
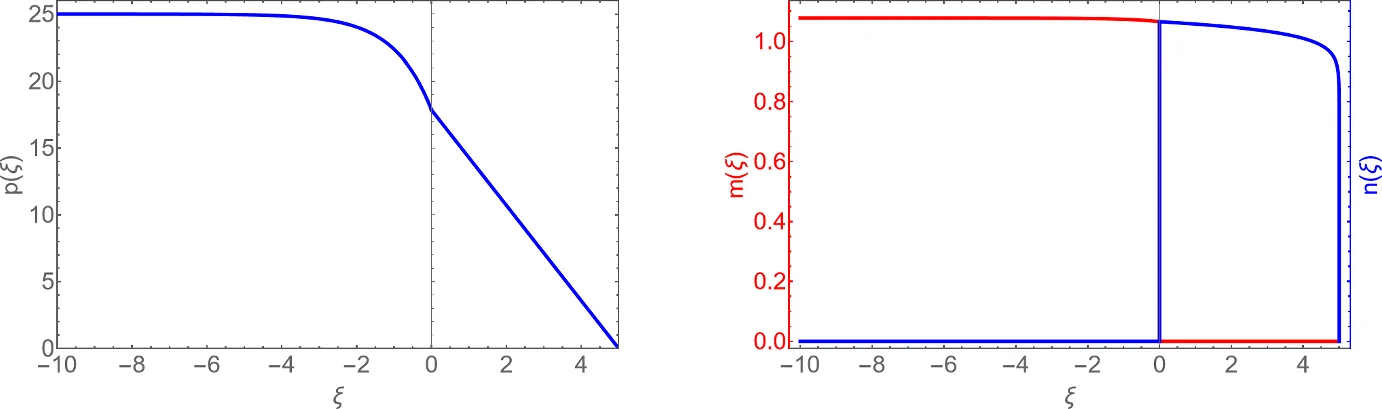
Left: Pressure fields from Eqs. (7, 8) for the travelling wave solution. Right: Corresponding cellular densities *m* and *n*. All curves are obtained setting r=5, $P_M=25$, μ=1, ν=2 and γ=30

The travelling wave solution is illustrated in Fig. 1, showing the pressure fields in Eqs. (7, 8) (left) and the corresponding cellular densities *m* and *n* (right). As already noted in Ref. (Lorenzi et al., 2017), the one-dimensional dynamical selection of the travelling wave depends on the ratio $\mu /\nu$. Since μ and ν are respectively the mobilities of the proliferating and non-proliferating populations, $\mu <\nu$ means that the proliferating cells are less mobile. In this case, the segregated travelling wave is dynamically selected and the non-proliferating population remains at the leading edge, as confirmed numerically in Fig. 6. For $\mu >\nu$, the proliferating population is more mobile and mixing between the populations is observed. This mobility dependence concerns one-dimensional dynamical selection, not the existence of the travelling wave. We next investigate whether the selected planar front remains stable to transverse perturbations or develops a transverse fingering instability (Saffman and Taylor 1958).

**Fig. 2:**
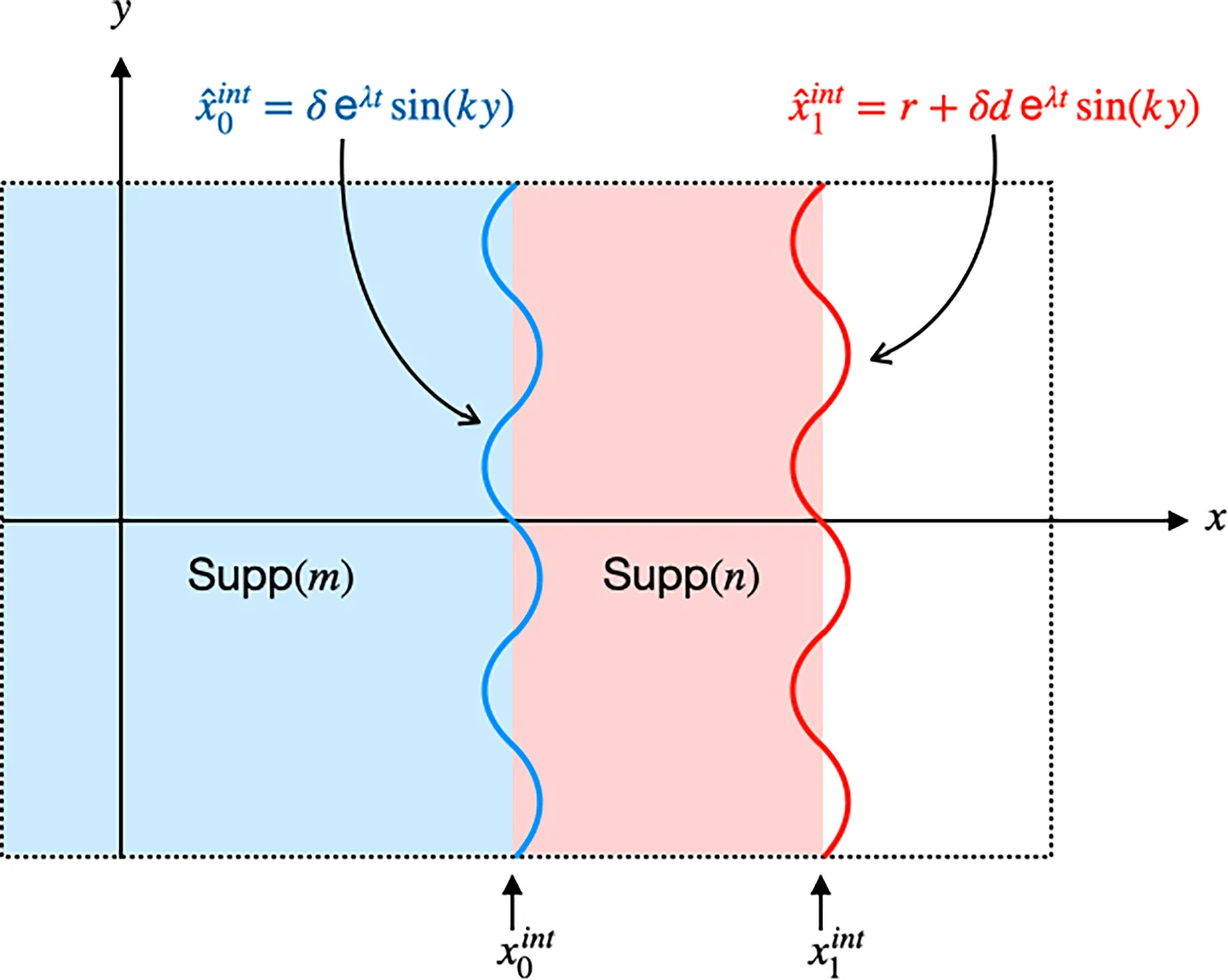
Sketch of the perturbed domains: δ is the small amplitude of the interface perturbation, while $d \ \delta$ is the amplitude of the perturbation of the free surface of n. Both perturbations have a wavenumber *k* along the vertical direction *y* with a characteristic growth rate λ

## Two-dimensional linear stability analysis of a travelling planar front

To study the stability of the travelling wave solution derived in Sect. 2.2, we apply a small perturbation to the planar front dividing the two populations of cells, as shown in Fig. 2. Let $x_0^{int}$ be the position of the planar interface front between *m* and *n* and $x_1^{int}$ be the position of the free surface of *n*. At initial time t=0 we assume $x_0^{int}=0$ and, accordingly, $x_1^{int}=r$. We then consider a sinusoidal perturbation of $x_0^{int}$, so the position of the perturbed interface in the (*x*, *y*) plane reads

11a $$\begin{aligned} \hat{x}_0^{int} = \sigma t + \delta \text {e}^{\lambda \, t} \, \sin (k \, y) \, , \end{aligned}$$

where δ>0 is a small number, *k* is the wavenumber, and $\lambda = \lambda (k)$ is the characteristic growth rate of each *k* mode. For physical consistency, recalling that *n* lies on a compact support, the ansatz for $x^{int}_1$ is taken in the same form, as follows

11b $$\begin{aligned} \hat{x}^{int}_1 =r + \sigma t + \delta \, d\, \text {e}^{\lambda \, t} \, \sin (k \, y) \, , \end{aligned}$$

where *d* is a constant, to be determined through boundary conditions. According to (11), the perturbed interfaces have the following perturbed velocities:

12 $$\begin{aligned} \hat{\sigma }_0 = \frac{\text {d} \hat{x}_0^{int} }{ \text {d} t} = \sigma + \delta \, \lambda \, \text {e}^{\lambda \, t} \, \sin (k \, y) \qquad \text {and} \qquad \hat{\sigma }_1 = \frac{\text {d} \hat{x}_1^{int} }{ \text {d} t} = \sigma + \delta \, d \, \lambda \, \text {e}^{\lambda \, t} \, \sin (k \, y) , \end{aligned}$$

and, consequently

$$ \hat{\sigma }_1 - \hat{\sigma }_0 = (d-1) \, \delta \, \lambda \, \text {e}^{\lambda \, t} \, \sin (k \, y) \,. $$

The value of d thus quantifies the relative perturbation amplitudes of the two boundaries. In particular, if λ>0 and d<1, the perturbation velocity amplitude of the internal interface is larger than that of the free surface, consistently with the onset of fingering.

To perform a stability analysis, we assume a perturbed pressure field in the same functional form, as follows:

13a $$\begin{aligned} p (x,y,t) = p^0_m(\xi ) + \delta \, \hat{p}_m (\xi ) \, \text {e}^{\lambda \, t} \, \sin (k \, y) \qquad&\text {in} \qquad \text {Supp} (m) \, , \end{aligned}$$

13b $$\begin{aligned} p (x,y,t) = p^0_n(\xi ) + \delta \, \hat{p}_n (\xi ) \, \text {e}^{\lambda \, t} \, \sin (k \, y) \qquad&\text {in} \qquad \text {Supp} (n) \, , \end{aligned}$$

with $p^0_n$ and $p^0_m$ indicating the pressure obtained in the one-dimensional wave front solutions, i.e. Equations (7) and (8), respectively. As before, we study the perturbed solutions in $\text {Supp}(n)$ and $\text {Supp}(m)$ separately.

In $\text {Supp}(n)$, we substitute Eq. (13b) into (1b). At the first order in δ, we obtain:

$$ \left[ \, \lambda + \gamma k^2 \sigma (r-\xi ) \, \right] \hat{p}_n (\xi ) + \sigma \left[ \, \hat{p}_n'(\xi ) + \gamma (\xi -r) \hat{p}_n''(\xi ) \, \right] = 0 \,. $$

This equation can be simplified in the incompressible limit $\gamma =1/\,\varepsilon \rightarrow \infty$, yielding

$$ k^2 \hat{p}_n(\xi ) - \hat{p}_n''(\xi ) + \mathcal {O}(\,\varepsilon ) = 0 \,, $$

where the incompressible limit is taken at fixed k>0 and fixed spectral parameter λ=O(1). Thus, the solution $\hat{p}_n(\xi )$ reads:

14 $$\begin{aligned} \hat{p}_n (\xi ) = c_3 \text {e}^{k \xi } + c_4 \text {e}^{- k \xi } \, \qquad \text {in} \qquad \text {Supp}(n) \, , \end{aligned}$$

where $c_3$ and $c_4$ are integration constants. These can be determined by requiring the velocity of the *n*-population on $\hat{x}^{int}_0$ to match $\hat{\sigma }_0$, and that *n* vanishes on $\hat{x}^{int}_1$. Consequently, we enforce

$$ - \nu \, \, \left. \frac{\text {d} p}{\text {d} x} \right| _{\hat{x}^{int}_0} = \hat{\sigma }_0 \qquad \text {and} \qquad n (\hat{x}^{int}_1) = 0 \,, $$

which, at the first order of δ, gives:

$$ \hat{p}'_n (0) + p_n^0{\prime \prime } (0) = -\frac{\lambda }{\nu } \qquad \text {and} \qquad \hat{p}_n (r) + d \, p_n^0{\prime } (r) = 0 \,, $$

so that:

$$ c_3= -\frac{\lambda -d k \sigma \text {e}^{k r}}{k \nu +k \nu \text {e}^{2 k r}} \qquad \text {and} \qquad c_4 = \frac{d k \sigma \text {e}^{k r}+\lambda \text {e}^{2 k r}}{k \nu +k \nu \text {e}^{2 k r}} \,. $$

We now solve the problem in $\operatorname {Supp}(m)$. Substituting (13a) into the governing equation for *m* and linearizing at first order in δ, we obtain

$$ \left( 1 + k^2 \, \mu \right) \hat{p}_m(\xi )- \, \mu \, \hat{p}_m''(\xi ) + \mathcal {O}(\,\varepsilon ) = 0 \,, $$

also assuming the incompressible limit $\gamma \rightarrow \infty$. Its solution yields

15 $$\begin{aligned} \hat{p}_m (\xi ) = c_5 \text {e}^{\frac{\xi \sqrt{k^2 \mu +1}}{\sqrt{\mu }}}+ c_6 \text {e}^{-\frac{\xi \sqrt{k^2 \mu +1}}{\sqrt{\mu }}} \qquad \text {in} \qquad \text {Supp}(m) \, , \end{aligned}$$

where the integration constants $$c_5$$ and $$c_6$$ can be determined by imposing $p^{\prime }\left(-\infty \right)=0$ along with the continuity condition for *p* across $$\hat{x}^{int}_0$$. At the first order of δ, such conditions read

$$ \hat{p}'_m ( -\infty ) = 0 \qquad \text {and} \qquad \hat{p}_m (0^-) + p_m^0{\prime } (0^-) = \hat{p}_n (0^+) + p_n^0{\prime } (0^+) \,, $$

so we get

$$ c_5=\frac{1}{k \nu } \left( d \, k \, \sigma \, \text {sech}(k r){+}\frac{k \nu P_M {-} k \sigma \left( \sqrt{\mu }{+}r\right) }{\sqrt{\mu }}+\lambda \tanh (k r) \right) \qquad \text {and} \qquad c_6 = 0 \,. $$

To calculate *d*, we apply the compatibility condition for the velocity of *n* on the free surface $\hat{x}^{int}_1$, that is:

$$ - \nu \,\, \left. \frac{\text {d} p}{\text {d} x} \right| _{x=\hat{x}^{int}_1} = \hat{\sigma }_1 \,. $$

At the first order of δ and for $\gamma \rightarrow \infty$, we get:

$$ \hat{p}'_n (r) + p_n^0{\prime \prime } (r) = -\frac{\lambda \, d}{\nu } \,, $$

which gives:

$$ d = \frac{\lambda }{\lambda \cosh (k r)+k \sigma \sinh (k r)} \,. $$

The coefficient *d* is the ratio between the perturbation amplitude of the external free boundary and that of the internal interface. Since $\lambda _1(k)>0$ on the unstable branch,

$$ 0<d= \frac{\lambda _1(k)}{\lambda _1(k)\cosh (kr)+k\sigma \sinh (kr)}<\frac{1}{\cosh (kr)}<1. $$

The two boundaries therefore have the same temporal growth rate, but the free-boundary perturbation has a smaller amplitude. This attenuation results from the transmission of the incremental pressure field across the non-proliferating layer.

Finally, we are left to compute the dispersion relation giving λ as a function of *k*. To do so, we apply the compatibility equations for the perturbed interface velocity. The latter extends the one-dimensional matching condition, imposing continuity of the mobility-weighted normal pressure gradient at $\hat{x}^{int}_0$, as follows:

$$ \mu \, \left. \frac{\text {d} p}{\text {d} x} \right| _{x=\hat{x}^{int -}_0} = \nu \, \left. \frac{\text {d} p}{\text {d} x} \right| _{x=\hat{x}^{int +}_0} \,, $$

which, after substitution of (13), gives at the first order of δ:

$$ \mu \left[ p_m^0 {\prime \prime }(0^-) + \hat{p}_m' (0^-) \right] = \nu \left[ p_n^0 {\prime \prime }(0^+) + \hat{p}_n' (0^+) \right] \,. $$

By substituting (7), (8), (14), and (15), along with the calculated expressions for $c_3$, $c_4$, $c_5$, $c_6$, *d* and σ, we finally obtain:

$$\begin{aligned}\begin{gathered} \frac{1}{k \nu \left( \nu +\sqrt{\mu } r\right) \left( k \sqrt{\mu } \nu P_M \sinh (k r)+\lambda \cosh (k r) \left( \nu +\sqrt{\mu } r\right) \right) } \\ \left[ \sqrt{\mu } \sinh (k r) \left( -\nu ^2 \left( \lambda ^2 \sqrt{k^2 \mu +1} \right. \right. \right. \\ \left. \left. \left. -k^2 P_M^2 \left( \sqrt{k^2 \mu +1} (\mu -\nu )+\nu \right) +\lambda k^2 \nu P_M\right) -\lambda \sqrt{\mu } \nu r \left( 2 \lambda \sqrt{k^2 \mu +1}+k^2 \nu P_M\right) \right. \right. \\ \left. \left. +\lambda ^2 \mu \left( -r^2\right) \sqrt{k^2 \mu {+}1}\right) {-}k \lambda \nu \cosh (k r) \left( \nu {+}\sqrt{\mu } r\right) \right. \\ \qquad \left. \left( \nu \left( P_M \left( \sqrt{k^2 \mu {+}1}{-}1\right) {+}\lambda \right) {+}\lambda \sqrt{\mu } r\right) \right] = 0 \,. \end{gathered}\end{aligned}$$

The former admits the following solutions:

16 $$\begin{aligned} \lambda _{1,2} = \frac{ k P_M \nu }{2 \left( \nu +\sqrt{\mu } r\right) \left( \sqrt{\mu } \sqrt{k^2 \mu +1} \sinh (k r)+k \nu \cosh (k r)\right) } \left( A \pm \sqrt{B} \right) \, , \end{aligned}$$

with

$$ A=\nu \cosh (kr)-\nu \sqrt{1+k^2\mu }\cosh (kr)-k\sqrt{\mu }\,\nu \sinh (kr), $$

and

$$\begin{aligned} B&= 4 \mu \left( k^2 \mu +1\right) (\mu -\nu ) \sinh ^2(k r)+\nu \left( -\sqrt{k^2 \mu +1} (2 \mu +\nu ) \right. \\&\quad + \left. k \sqrt{\mu } \left( \sqrt{k^2 \mu +1} (2 \mu -\nu )+\nu \right) \sinh (2 k r)+\nu \right) \\&\quad + \nu \left( k^2 \mu \nu -\nu \sqrt{k^2 \mu +1}+2 \mu \sqrt{k^2 \mu +1}+\nu \right) \cosh (2 k r) \,. \end{aligned}$$

In the range of interest $\nu >\mu$, the planar travelling wave is unstable to transverse perturbations over a finite band of wavenumbers. From (16), $\lambda _1(k)\rightarrow 0$ as k→0. The short-wave decay can be made explicit:

$$ \lambda _1(k) = -\frac{P_M\nu \sqrt{\mu }(\nu -\mu )}{(\nu +r\sqrt{\mu })(\nu +\mu )}\,k +\mathcal {O}(1) \qquad \text {as }k\rightarrow \infty . $$

Thus, high-wavenumber perturbations are strongly damped even though no interfacial tension is included. The large-*k* damping results from the spatial localization of the incremental pressure fields and from the velocity and pressure matching conditions at the two moving boundaries.

The second spectral branch is always stable. Indeed, writing the compatibility condition as $a_k\lambda ^2+b_k\lambda +c_k=0$, one has $a_k<0$ and $b_k<0$ for every k>0. The two roots are real and satisfy $\lambda _1+\lambda _2=-b_k/a_k<0$. Since $\lambda _1\ge \lambda _2$, it follows that $\lambda _2(k)<0$ for every k>0; hence only $\lambda _1$ can generate the instability.

The present instability shares with Saffman–Taylor fingering the amplification of interfacial perturbations under Darcy transport and the coupling of pressure disturbances across a layer of finite width. The destabilizing mobility ordering is, however, reversed. Classical Saffman–Taylor fingering occurs when the displacing phase is more mobile than the displaced one, whereas here the segregated front becomes unstable for $\nu >\mu$, with the proliferating population being the less mobile phase. This reversal reflects the different driving mechanism: the front velocity is generated internally by volumetric proliferation and the associated pressure field rather than imposed by an external displacement. The Saffman–Taylor analogy therefore concerns the Darcy interfacial structure, reflected here by the factors sinh(kr) and cosh(kr), while the instability mechanism is intrinsically growth-driven. The short-wave behaviour is also different: in the absence of surface tension classical Saffman–Taylor growth is unbounded as $k\rightarrow \infty$, whereas the present problem has the intrinsic decay $\lambda _1(k)\sim -Ck$, with C>0.

**Fig. 3:**
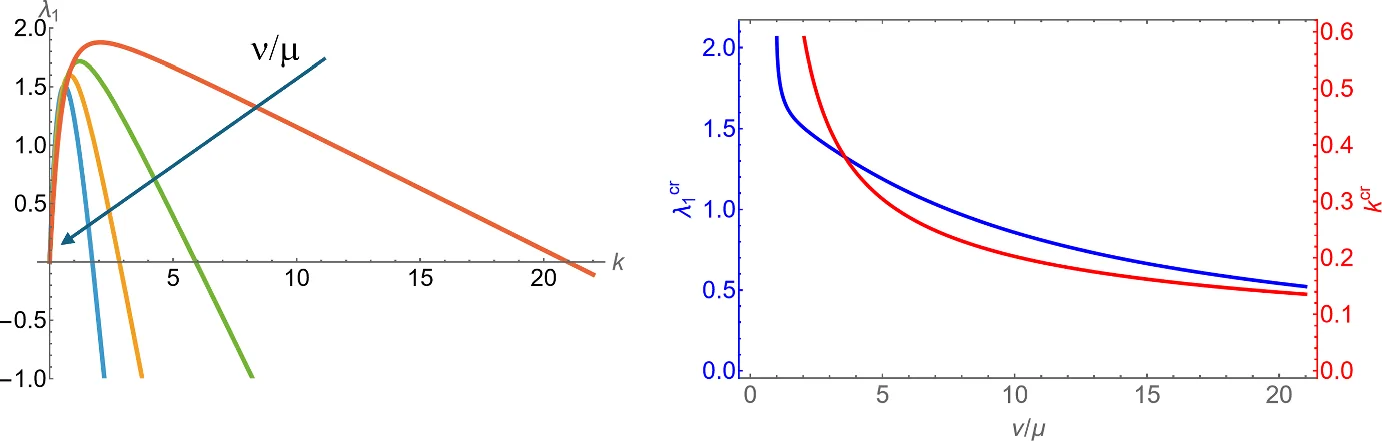
Left: dispersion curves $\lambda _1$ versus *k* in the regime $\mu <\nu$, for $\nu /\mu =1.05,1.2,1.5,2$, with the arrow indicating $\nu /\mu$ increasing. Right: most unstable wavenumber $k^{cr}$ and corresponding growth rate $\lambda _1^{cr}$ as functions of $\nu /\mu$. All curves are obtained with r=5, $P_M=25$, and μ=1

The dispersion curves in Fig. 3 correspond explicitly to $\mu <\nu$, with μ=1 and $\nu /\mu =1.05,1.2,1.5,2$. They exhibit a positive maximum $\lambda _1^{cr}$ at a finite wavenumber $k^{cr}$.

**Fig. 4:**
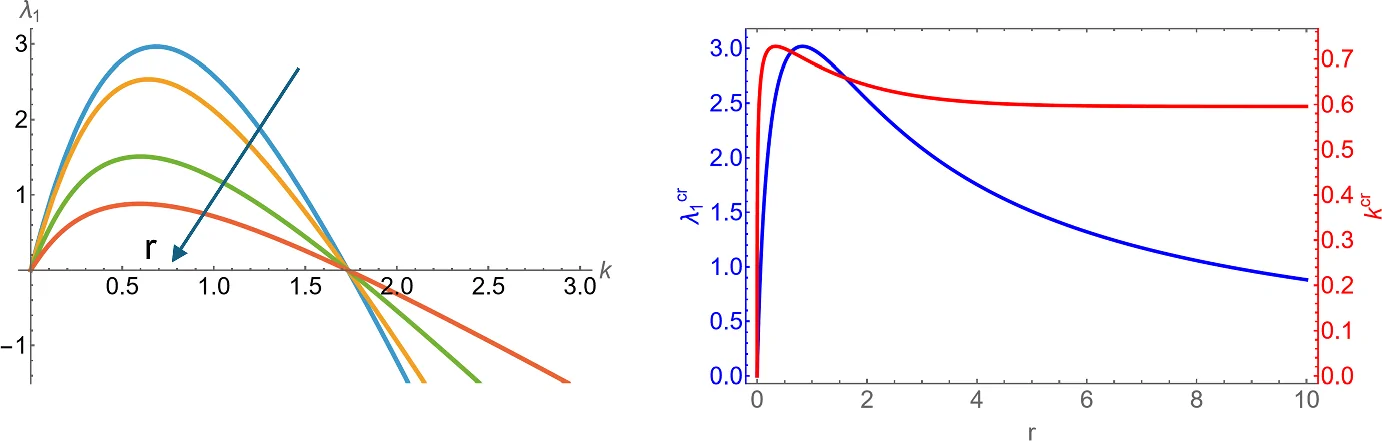
Left: Dispersion curves $\lambda _1$ versus *k* for r=1.1 (blue), 2 (yellow), 5 (green), and 10 (red). Right: most unstable wavenumber $k^{cr}$ (red solid line) and the corresponding growth eigenvalue $\lambda _1^{cr}$ (blue solid line) as a function of *r*. All curves are obtained setting $P_M=25$, μ=1 and ν=2 (color figure online)

Similarly, Fig. 4 shows the dispersion curves $\lambda _{1}^{K}$ for varying *r*, setting v=2 and μ = 1. In this case, for small *r*, both the most unstable wavenumber $k^{cr}$ and the corresponding growth rate $\lambda _1^{cr}$ increase as the finite support size *r* of the healthy phase increases, until a critical size in the range of r≈1, after which the trend is reversed.

**Fig. 5:**
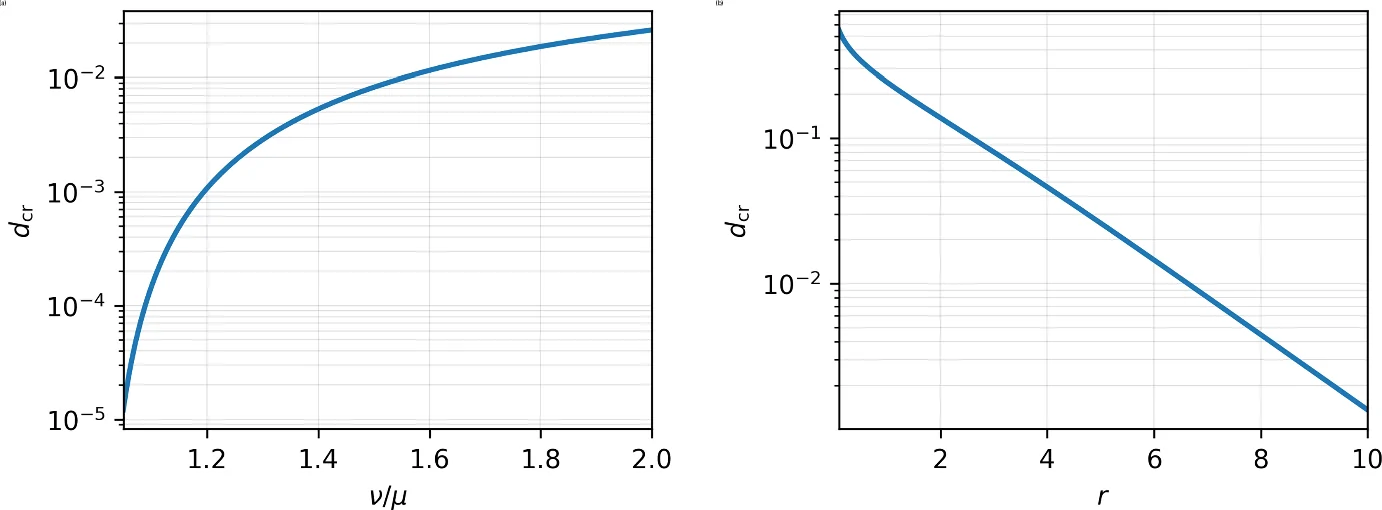
Coefficient *d* evaluated at the most unstable mode. Left: dependence on $\nu /\mu$ for r=5, $P_M=25$, and μ=1. Right: dependence on *r* for $P_M=25$, μ=1, and ν=2. The vertical axes are logarithmic

Figure 5 shows *d* evaluated at the most unstable mode, by setting $k=k^{cr}$ and $\lambda _1=\lambda _1^{cr}$. At fixed r=5, *d* remains small throughout the considered range of mobility ratios. At fixed $\nu /\mu =2$, *d* decreases rapidly with *r*. For the parameters used in the nonlinear simulations, d=0.242 for r=1 and d=0.026 for r=5.

## Numerical solution

In this section, we present a robust, positivity–preserving numerical scheme for solving the coupled nonlinear advection–diffusion-reaction system given by Eq. (1) and we illustrate the resulting numerical solutions in one-dimensional and two-dimensional domains.

### Numerical methods

To solve the coupled nonlinear system (1) we construct a numerical strategy designed around three requirements imposed by the regime considered here: preservation of non-negative cell densities, conservative resolution of the strongly coupled pressure-driven transport, and robustness for large pressure exponents γ, when sharp interfaces develop. These requirements motivate a monolithic backward-Euler discretization combined with a projected Newton–Krylov solver, rather than an operator-splitting treatment of transport and reaction.

The method is based on a reformulation of (1) as a fully nonlinear cross–diffusion system which is then discretized in space via Centered Finite Differences (CFD) on a uniform Cartesian grid (with a suitable averaging of the nonlinear diffusion coefficients) and advanced in time via backward–Euler (BE) time stepping.

The resulting sequence of nonlinear algebraic systems is solved by means of a globalized projected Newton–Krylov algorithm (Chen and Vuik 2017) in order to enforce the non-negativity constraints $m \ge 0,\; n \ge 0$ throughout the solution procedure.

#### Space-Time discretization and nonlinear residual

To describe our numerical strategy, we start by rewriting (1) as follows

17 $$\begin{aligned} {\left\{ \begin{array}{ll} \dfrac{\partial m}{\partial t} - \textrm{div} \left( \widetilde{\mu }(m,n) \nabla m\right) - \textrm{div} \left( \widetilde{\mu }(m,n) \nabla n\right) = G(m,n) m \\ \dfrac{\partial n}{\partial t} - \textrm{div} \left( \widetilde{\nu }(m,n) \nabla n\right) - \textrm{div} \left( \widetilde{\nu }(m,n) \nabla m\right) = 0. \end{array}\right. } \end{aligned}$$

where G(m,n):=G(p(m,n)).

18 $$\begin{aligned} {\left\{ \begin{array}{ll} \widetilde{\mu }(m, n) = \mu \,m (\gamma +1) (n+m)^{\gamma -1}\\ \widetilde{\nu }(m, n) =\nu \, n(\gamma +1) (n+m)^{\gamma -1}\\ \end{array}\right. } \end{aligned}$$

For the sake of simplicity, we henceforth consider (17)–(18) to be solved in a one–dimensional bounded spatial domain $\Omega \equiv \left( 0,L\right)$ that we discretize as a set of uniformly spaced points $\Omega _h \equiv \left\{ x_i\right\} _{i=0}^{N_h}$ with $h=\dfrac{L}{N_h}$ and $x_i = i \cdot h$. The extension to the two–dimensional case on a tensor-product grid is straightforward. Let $t^k$ be the discrete time levels and $\Delta t^k = t^{k+1}-t^k$ and denote by $n^{k}_i, m^{k}_i$ the approximations to the unknown functions *m*, *n* at the *k*-th time step and *i*-th grid point. The fully discrete counterpart of  (17)–(18) then reads

19 $$\begin{aligned} \begin{aligned} m^{k+1}_{i} =\ &m^{k}_{i} + \dfrac{\Delta t^{k}}{h^2} \left[ \widetilde{\mu }^{k+1}_{i-\frac{1}{2}} \left( m^{k+1}_{i-1} - m^{k+1}_{i} \right) + \widetilde{\mu }^{k+1}_{i+\frac{1}{2}} \left( m^{k+1}_{i+1} - m^{k+1}_{i} \right) \right] \\&+\dfrac{\Delta t^{k}}{h^2} \left[ \widetilde{\mu }^{k+1}_{i-\frac{1}{2}} \left( n^{k+1}_{i-1} - n^{k+1}_{i} \right) + \widetilde{\mu }^{k+1}_{i+\frac{1}{2}} \left( n^{k+1}_{i+1} - n^{k+1}_{i} \right) \right] + \Delta t^{k} G^{k+1}_i m^{k+1}_i \\ n^{k+1}_{i} =\ &n^{k}_{i} + \dfrac{\Delta t^{k}}{h^2} \left[ \widetilde{\nu }^{k+1}_{i-\frac{1}{2}} \left( n^{k+1}_{i-1} - n^{k+1}_{i} \right) + \widetilde{\nu }^{k+1}_{i+\frac{1}{2}} \left( n^{k+1}_{i+1} - n^{k+1}_{i} \right) \right] \\&+\dfrac{\Delta t^{k}}{h^2} \left[ \widetilde{\nu }^{k+1}_{i-\frac{1}{2}} \left( m^{k+1}_{i-1} - m^{k+1}_{i} \right) + \widetilde{\nu }^{k+1}_{i+\frac{1}{2}} \left( m^{k+1}_{i+1} - m^{k+1}_{i} \right) \right] \end{aligned} \end{aligned}$$

where the following *upwind* averaging of the (cross–)diffusion coefficient $\widetilde{\mu }$ is used

20 $$\begin{aligned} \widetilde{\mu }^{k+1}_{i-\frac{1}{2}} = {\left\{ \begin{array}{ll} \widetilde{\mu } (m^{k+1}_{i-1}, n^{k+1}_{i-1}) \quad \text{ if } p(m^{k+1}_{i-1}, n^{k+1}_{i-1}) > p(m^{k+1}_{i}, n^{k+1}_{i}) \\ \widetilde{\mu } (m^{k+1}_{i}, n^{k+1}_{i}) \quad \text{ otherwise } \end{array}\right. } \end{aligned}$$

and a similar approach is taken for $\widetilde{\nu }$. Collecting all the discrete unknowns in a vector

$$U^{k+1} = \left[ m^{k+1}_0, \ldots , m^{k+1}_{N_h}, n^{k+1}_0, \ldots , n^{k+1}_{N_h}\right] ^T,$$

the implicit step (19) can be written compactly as

21 $$\begin{aligned} F(U^{k+1}) \equiv U^{k+1}-U^k-\Delta t^k\;\mathcal {R}(U^{k+1}) = 0, \end{aligned}$$

where $\mathcal {R}$ denotes the spatial discretization of the flux and reaction operators.

#### Projected Newton–Krylov solver with projected-gradient safeguard

At each time step, we solve (21) with a projected Newton–Krylov method mixed with a projected-gradient direction, following the globalization framework in Chen and Vuik (2017). Let $P:\mathbb {R}^N\rightarrow \mathbb {R}^N_+$ denote the component–wise projection $(P(z))_i = \max \{z_i,0\}.$ Starting from a feasible initial guess $U^{k+1,0}\ge 0$, we generate iterates

22 $$\begin{aligned} U^{k+1,j+1}=P\!\left( U^{k+1,j}+\lambda _j d_j\right) , \end{aligned}$$

where $d_j$ is either a projected Newton direction or a projected-gradient direction, and $\lambda _j\in (0,1]$ is chosen by backtracking.

When a Newton step is attempted, $d_j$ is computed by an *inexact Newton* condition (Eisenstat and Walker 1996; Kelley 2003): we approximately solve $\textrm{d}F(U^{k+1,j})\,d_j = -F(U^{k+1,j})$ so that

23 $$\begin{aligned} \Vert F(U^{k+1,j}) + \textrm{d}F(U^{k+1,j})\ d_j\Vert \le \eta _j \Vert F(U^{k+1,j})\Vert , \end{aligned}$$

with $\textrm{d}F$ the Jacobian (or a consistent approximation) and $\eta _j\in [0,\eta _{\max })$ a forcing term. The linearized systems are solved with a Krylov subspace method, namely the generalized minimal residual method (GMRES) (Saad and Schultz 1986). We update the forcing term $\eta _j$ according to Eisenstat and Walker (1996),

24 $$\begin{aligned} \eta _{j+1}=\min \!\Bigl (\eta _{\max },\;\Gamma _\eta \Bigl (\frac{\Vert F(U^{k+1,j+1})\Vert }{\Vert F(U^{k+1,j})\Vert }\Bigr )^{\alpha _\eta }\Bigr ), \end{aligned}$$

where $\alpha _\eta >1$ and $\Gamma _\eta \in (0,1]$ are algorithmic parameters (here we take $\alpha _\eta =2$ and $\Gamma _\eta =1$), together with the standard safeguards described in Kelley (2003).

To globalize convergence under projection, we use a geometric backtracking. For the projected Newton step we set $\lambda _j=\beta ^{\,l}$, $l=0,1,\dots ,l_{\max }$, with $\beta \in (0,1)$, and we accept the projected iterate (22) if the sufficient decrease condition (Chen and Vuik 2017)

25 $$\begin{aligned} \bigl \Vert F(P(U^{k+1,j}+\lambda _j d_j))\bigr \Vert \le \Bigl (1 - t_{ls}\,\lambda _j\ (1-\eta _j)\Bigr )\,\Vert F(U^{k+1,j})\Vert \end{aligned}$$

holds.

If no acceptable $\lambda _j$ is found within $l_{\max }$ reductions, we switch to a projected-gradient direction for the merit function $\Theta (U)=\tfrac{1}{2}\Vert F(U)\Vert ^2$ (Calamai and Moré 1987; Chen and Vuik 2017). In this case we backtrack with $\lambda _j = \beta _{G}^{l}$, $l=0,1,\dots ,l_{\max }$, with $\beta _G\in (0,1)$, until the Armijo condition holds:

26 $$\begin{aligned} \Theta \!\bigl (P(U^{k+1,j}+\lambda _j d_j)\bigr ) \le \Theta (U^{k+1,j}) + \sigma _\eta \,\nabla \Theta (U^{k+1,j})^{T}\!\Bigl (P(U^{k+1,j}+\lambda _j d_j)-U^{k+1,j}\Bigr ), \end{aligned}$$

where $\sigma _\eta \in (0,1)$ is the Armijo parameter. We terminate the nonlinear iterations when $\Vert F(U^{k+1,j})\Vert \le 10^{-8}$ or, when $\Vert U^{k+1,j+1}-U^{k+1,j}\Vert \le 10^{-6}$. In the tests shown below, we use the parameter set $t_{ls}=\sigma _\eta =10^{-4},\; \eta _{\max }=0.9,\; l_{\max }=10,\; \beta =0.5,\; \beta _G=0.8.$

#### Adaptive time–step selection

We complement backward Euler with a first-order adaptive controller based on local extrapolation (Hairer et al. 1987). Given accepted solutions $U^{k}$ and $U^{k-1}$, we build a predictor (used also as initial guess for the nonlinear solve) by linear extrapolation, $U^{k+1,0}=U^k + \frac{\Delta t^k}{\Delta t^{k-1}}(U^k-U^{k-1}).$ After computing $U^{k+1}$, we estimate the local defect as $err=\Vert U^{k+1}-U^{k+1,0}\Vert .$ If $err < \texttt {tol}_t$ (we take $\texttt {tol}_t=10^{-3}$), the step is accepted and the next time step is updated as

27 $$\begin{aligned} \Delta t^{k+1} = \Delta t^{k}\,\min \!\Bigl (\texttt {fac}_{\max },\max \!\bigl (\texttt {fac}_{\min }, \texttt {fac}\,\sqrt{\texttt {tol}_t/err}\bigr )\Bigr ), \end{aligned}$$

with standard safety factors $\texttt {fac}\in (0,1)$ and bounds $\texttt {fac}_{\min }<1<\texttt {fac}_{\max }$. If $err\ge \texttt {tol}_t$, the step is rejected and recomputed with $\Delta t^{k}\leftarrow \Delta t^{k}/2$. All simulations (1D and 2D) employ the adaptive time-step controller of Sect. 4.1, initialized with $\Delta t^{0}=10^{-5}$.

The present strategy differs from structure-preserving operator-splitting methods developed for reaction–cross-diffusion systems (Carrillo et al. 2018). In those approaches, transport and reaction are advanced separately, and a finite-volume discretization of the transport step provides discrete mass conservation and, in the symmetric gradient-flow setting, energy decay. Here, backward Euler time integration is applied monolithically to the coupled transport, cross-diffusion and reaction terms. The flux-difference form of the spatial discretization preserves the mass of the non-proliferating population and the reaction-driven mass balance of the proliferating population, up to the nonlinear-solver tolerance, while the projected Newton–Krylov iteration enforces non-negativity at the algebraic level. The method is designed primarily for robustness in the large-pressure-exponent regime and does not rely on, or claim, a discrete energy-dissipation property.

### Simulation results

First, we start by performing numerical simulations in a one-dimensional domain $\Omega _{1d}$, in order to validate the numerical results against the one-dimensional travelling wave solution given in Sect. 2. The domain $\Omega _{1d}=[0,30]$ is discretized with 150 points, while the initial time step is $10^{-5}$. Furthermore, we take the following initial conditions:

$$\begin{aligned} m(x,t=0):= a_m e^{-b_m x^2} \ \ and \ \ n(x,t=0) = a_n e^{-b_n (x-10)^2}, \end{aligned}$$

with $a_m = 0.1$, $b_m = 0.5$, $a_n = 0.8$ and $b_n = 0.5$. These initial conditions represent two distinct cell populations initially localized in separate spatial regions: population *m* is centered at x=0 with a small amplitude, while population *n* is centered at x=10 with a higher amplitude.

**Fig. 6:**
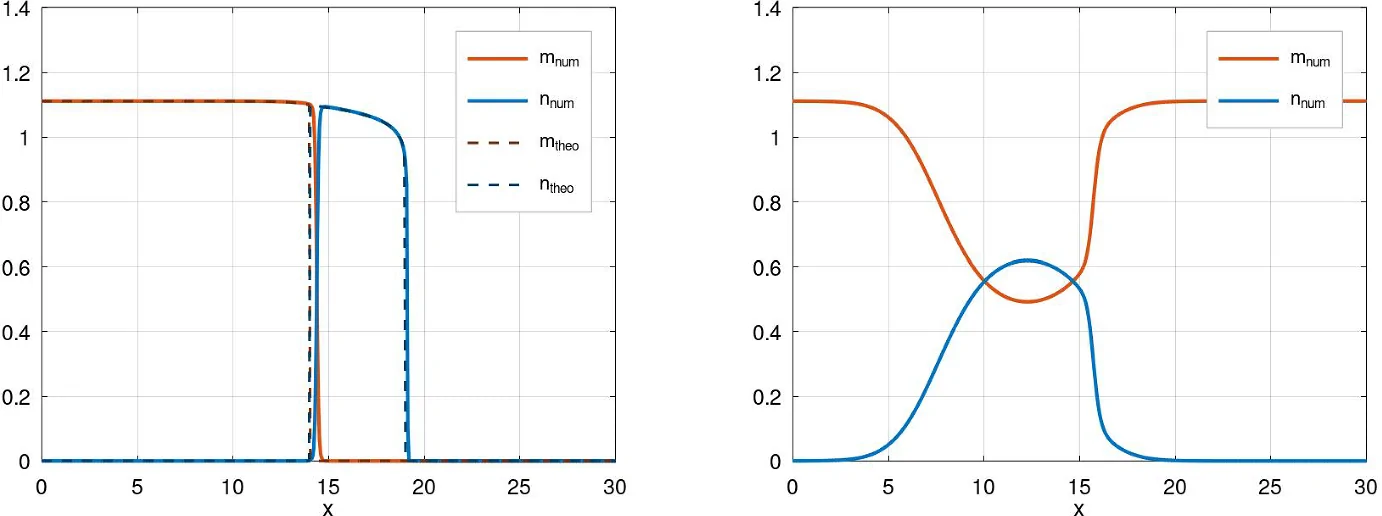
Comparison between the numerical and asymptotic one-dimensional travelling-wave solutions. The two mobility regimes $\mu <\nu$ (left, μ=1, ν=2) and $\mu >\nu$ (right, μ=2, ν=1) respectively produce a segregated front and population mixing. In the segregated case the numerical density profiles *n* and *m* (solid) are overlaid with the incompressible travelling-wave prediction (dashed). The theoretical profiles *n* and *m* are initialised with supports fixed by the front position of the numerical solution at t=0.6, by which time the front has fully formed and its propagation speed has reached the asymptotic value, and the two profiles are then compared at t=1.2

**Fig. 7:**
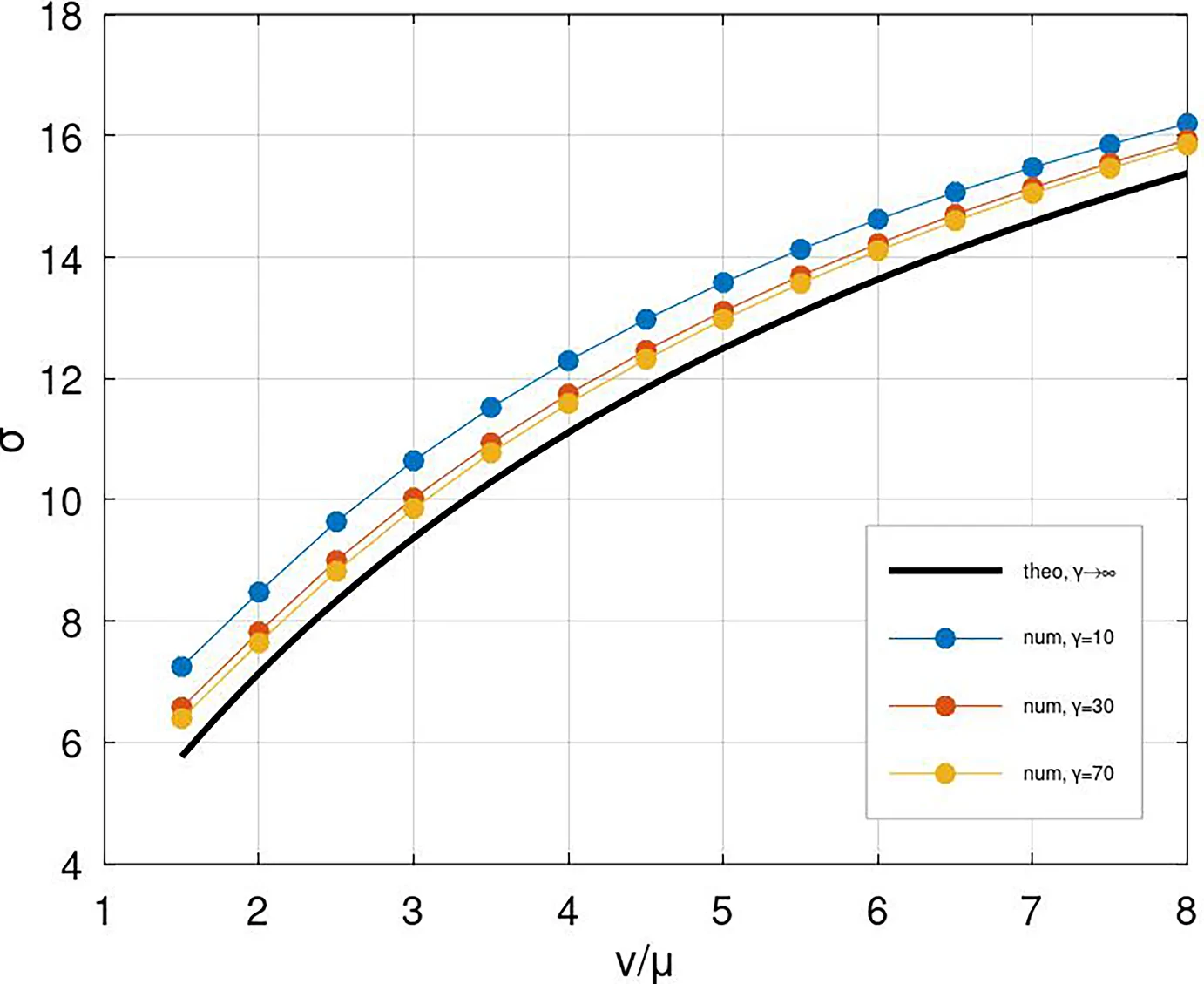
Measured propagation velocity σ as a function of $\nu /\mu$, compared with the asymptotic prediction of Eq. (10), for increasing values of γ. Already at γ=30, the value used throughout our simulations, the measured speed is close to the asymptotic limit. Here, σ is measured once the front has fully formed and its speed has settled onto the steady value

As shown in Fig. 6, the resulting dynamics is consistent with the theoretical expectations: we observe mixing of the two populations when $\mu > \nu$, and the formation of a travelling wave front composed of non-dividing cells remaining at the leading edge when $\mu < \nu$. The measured front velocity agrees with the value predicted by Eq. (10), and this agreement improves as γ increases, approaching the asymptotic limit (Fig. 7).

Second, we perform numerical simulations in a two-dimensional domain $\Omega _{2d}$, in order to investigate the morphological evolution of a planar wave. We consider the domain $\Omega _{2d} \equiv [-1, -1 +6\pi / k_{cr}] \times [-3 \pi / k_{cr}, 3 \pi / k_{cr}]$, where $k_{cr}$ is the most unstable wavenumber predicted by the linear stability analysis for the set of parameters under consideration. We consider a structured 2D mesh with 300 elements in each direction. We impose periodic boundary conditions in the transverse direction *y* and homogeneous no-flux conditions on the left/right boundaries in *x*. We consider a structured 300×300 grid. The variables *m*, *n*, and *p* are defined at the grid nodes, while the conservative fluxes are evaluated at the midpoints between neighbouring nodes in each coordinate direction.

Now we test the model considering a perturbed initial condition as derived in Sect. 3. For numerical ease, we introduce a smoother initial condition using a hyperbolic tangent regularization. To this end, we use the following initial condition:

$$\begin{aligned} m(0)&=\frac{1}{2}\left( \left( \frac{\gamma }{\gamma {+}1}[p_m^0(x){ + }\delta \hat{p}_m(x) \cos (k_{cr}y{+}\pi )]\right) ^{\frac{1}{\gamma }}\right) \left( 1{-}\tanh (\alpha (x {-} x_0^{int} ))\right) , \\ n(0)&= \frac{1}{4} \left( \left( \frac{\gamma }{\gamma +1}[p_n^0(x) + \delta \hat{p}_n(x) \cos (k_{cr}y+\pi )]\right) ^{\frac{1}{\gamma }}\right) \left( 1+\tanh (\alpha ( x-x_0^{int} ))\right) \\&\quad \times \left( 1-\tanh (\alpha ( x-x_1^{int}))\right) . \end{aligned}$$

The transitions are defined differently for the two populations, since the cancer cell population has just one boundary at $x_0^{int} = \delta \cos (k_{cr}y+\pi )$, while the non-dividing population has also a free boundary at $x_1^{int} = r+d \delta \cos (k_{cr}y+\pi )$. Thus, the parameter α determines the smoothness of the desired transition.

We start by investigating the dynamics of a travelling wave solution with r=1, μ=1, ν=2, $P_M=25$, γ=30, setting the perturbation amplitude δ=0.2. From the linear stability analysis, we derive the unstable growth rate $\lambda _{1}^{cr} = 0.1197 P_{M}$ and the most unstable wavenumber $k_{cr} = 0.6914$. We finally set the initial smoothing parameter as α=16 and an initial time step $10^{-5}.$

**Fig. 8:**
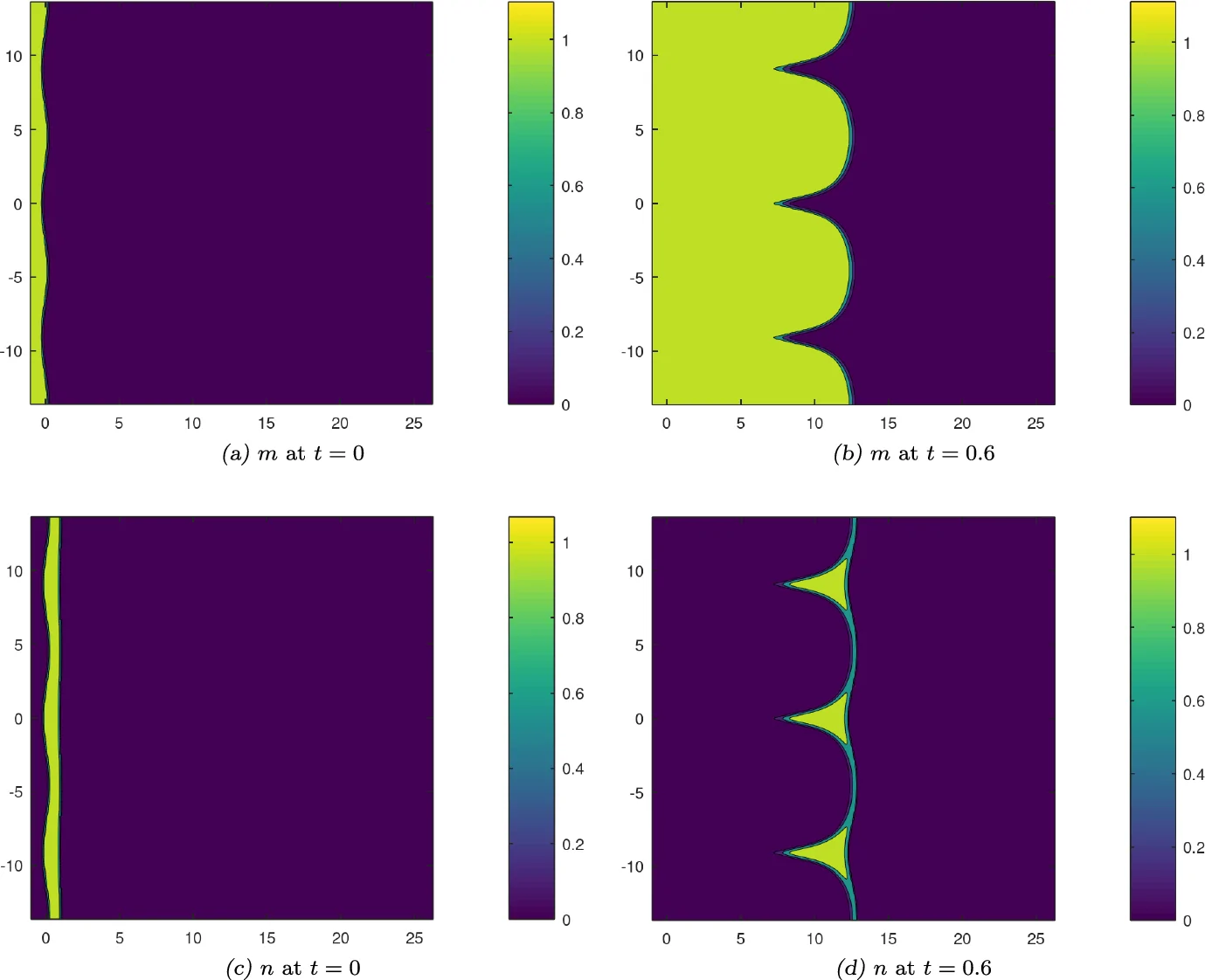
Numerical evolution of a travelling-wave solution with r=1, μ=1, v=2, $P_M = 25,$ γ=30. Starting from an initial perturbation of amplitude δ=0.2 in the fields *m* (**a**) and *n* (**c**), at t=0.6 the interface perturbation has grown in *m* (**b**) and *n* (**d**), indicating a morphological crossover towards fingering. See Ciarletta et al. (2026a, b) for an animated version of these plots

The simulation results are shown in Fig. 8.

**Fig. 9:**
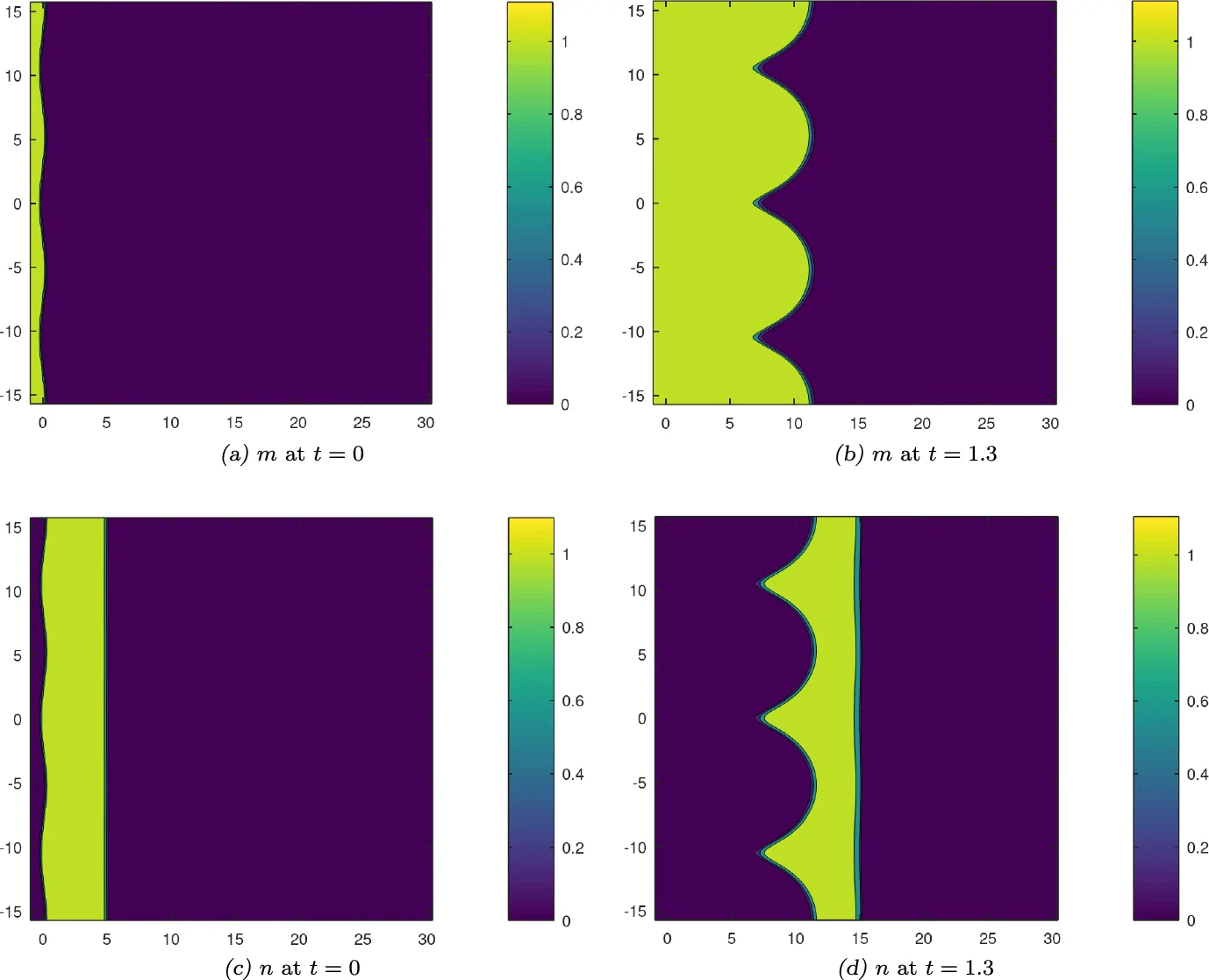
Numerical evolution of a travelling-wave solution with r=5, μ=1, ν=2, $P_M=25$, γ=30. Starting from an initial perturbation of amplitude δ=0.2 in the fields *m* (**a**) and *n* (**c**), at t=1.3 the interface perturbation has grown in *m* (**b**) and *n* (**d**) morphological crossover towards fingering. See Ciarletta et al. (2026a, b) for an animated version of these plots

We observe that the initial sinusoidal perturbation of the interface between the two populations grows over time, displaying a transition towards a finger-like shape. As the instability develops in the fully nonlinear regime, the front of *n* becomes very thin, which can eventually lead to numerical instabilities. Thus, we further considered an initial domain with larger support of *n*, setting r=5, and keeping the other parameters unchanged. From the linear stability analysis, for this case we compute the unstable growth rate $\lambda ^{cr}_1 = 0.06 \, P_M$ and the most unstable wavenumber $k_{cr} = 0.5993$. The simulation results are illustrated in Fig. 9.

The interface between the two populations develops a pronounced finger-like deformation, whereas the external free boundary remains comparatively smooth. This behavior is consistent with the attenuation factor d<1 predicted by the linear analysis.

To test quantitatively the growth predicted by the linear stability analysis, we track the peak-to-peak displacement of the internal interface,

$$ \Delta x(t) = \max _y x_{\textrm{int}}(y,t) - \min _y x_{\textrm{int}}(y,t). $$

For a single transverse mode in the linear regime, the analytical prediction is

$$ \Delta x(t) = \Delta x(0)e^{\lambda _1(k)t}. $$

**Fig. 10:**
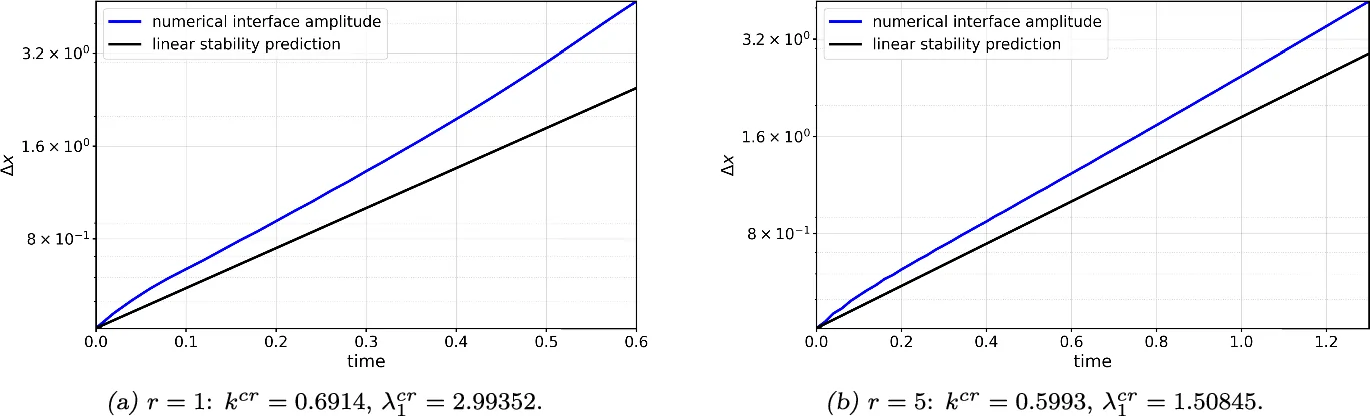
Numerical validation of the instability growth rate for μ=1, ν=2, $P_M=25$ and γ=30. The peak-to-peak displacement Δx(t) of the internal interface is shown on a logarithmic scale and compared with the incompressible linear prediction $\Delta x(0)\exp (\lambda _1^{cr}t)$. Panels (**a**) and (**b**) correspond respectively to r=1 and r=5 at their most unstable wavenumbers

Figure 10 compares this prediction with the numerical evolution at γ=30 for the two configurations considered above. For r=1, the most unstable mode has $k^{cr}=0.6914$ and $\lambda _1^{cr}=2.99352$, whereas for r=5, $k^{cr}=0.5993$ and $\lambda _1^{cr}=1.50845$. In both cases the numerical perturbation displays the exponential amplification predicted by the incompressible linear theory during the onset of the instability. Quantitative deviations remain at finite γ and become more pronounced as the perturbation leaves the linear regime. Together with the convergence of the travelling-wave velocity shown in Fig. 7, this provides a direct numerical validation of the incompressible predictions in the stiff-pressure regime.

We also quantify the possible departure of the base front from the semi-infinite travelling-wave solution caused by the finite computational domain and the finite value of γ. We define the mean interface position by

$$ \bar{x}_{\textrm{int}}(t) = \frac{1}{2} \left[ \max _y x_{\textrm{int}}(y,t) + \min _y x_{\textrm{int}}(y,t) \right] . $$

Over the time intervals displayed in the simulations, the average velocities $[\bar{x}_{\textrm{int}}(T)-\bar{x}_{\textrm{int}}(0)]/T$ are 16.99 for r=1 and 7.15 for r=5. The corresponding incompressible travelling-wave values from Eq. (10) are 16.67 and 7.14, respectively. Thus, the mean front velocity differs from the asymptotic value by approximately 2.0% for r=1 and approximately 0.14% for r=5 over the simulated time windows. The finite-domain and finite-compressibility drift of the base state is therefore small on the time scale used to characterize the onset of the transverse instability.

## Discussion and concluding remarks

This study combines analysis and computation to characterize transverse instabilities of planar travelling fronts in a two-population, pressure-driven model. The present analysis should be viewed as a planar travelling-wave counterpart to existing sharp-interface theories. Its novelty does not lie in identifying mobility contrast as a possible source of interfacial instability, which is already implicit in previous interface-dynamics results. Rather, it lies in making this mechanism explicit for a propagating segregated front. The normal-mode calculation gives the growth rate as a function of the transverse wavenumber, separates the stable and unstable branches, and quantifies how perturbations of the internal interface are coupled to the external free boundary. These spectral quantities are then tested against simulations of the finite-compressibility density model, thereby connecting the incompressible free-boundary prediction with the original reaction–cross-diffusion system.

We characterize here a transverse instability of the planar travelling front. It should not be confused with the existence of the one-dimensional travelling wave, which holds for general positive mobilities, nor with a rigorous one-dimensional stability theory. Our result shows that a front selected in the one-dimensional dynamics may still lose stability when two-dimensional transverse modes are allowed. The fingering instability identified here is related to Saffman–Taylor fingering through its Darcy-type interfacial structure, but differs in both its driving and its mobility criterion. In classical displacement, instability occurs when the displacing phase is more mobile than the displaced one (Daripa 2008). Here, the front is generated by proliferation and becomes unstable in the segregated regime $\nu >\mu$, so that the proliferating population is the less mobile phase. Proliferation-driven pressure generation therefore reverses the classical mobility criterion, while the finite-width layer retains the characteristic coupling of pressure perturbations between the two boundaries.

The pressure law and the growth law form a coupled constitutive choice. Here, the power-law relation $p=K_\gamma (m+n)^\gamma$ is combined with the pressure-dependent growth rate $G(p)=P_M-p$, whereas a logarithmic elastic stress arises in Recho et al. (2016b) from a different proliferation closure. The finite-compressibility travelling wave and its quantitative growth rates therefore depend on the constitutive law. In the incompressible limit, crowding imposes a local packing constraint, while proliferation remains pressure-dependent through *G*(*p*). The mobility-driven instability mechanism is consequently not specific to the power-law closure, although its dispersion spectrum may change under different constitutive assumptions.

Adhesion-mediated sorting and the instability studied here concern two distinct stages of the dynamics. A sufficiently large heterotypic interfacial tension can establish or maintain a sharp boundary between different cell populations (Canty et al. 2017). Starting from such a segregated configuration, our model isolates the effect of differential mobility on the pressure-driven stability of that boundary. An explicit interfacial tension is not included in the present formulation and is left for future work.

An analytical extension to large but finite γ requires an expansion in the two independent small parameters $\,\varepsilon =1/\gamma$ and δ. The order-$\,\varepsilon$ problem determines the correction to the travelling wave and its propagation speed, while the order-$\,\varepsilon \delta$ problem determines the first compressibility correction to the dispersion relation. The latter is a linear eigenvalue problem with variable coefficients and does not generally reduce to an explicit algebraic equation. Finite compressibility introduces quantitative corrections to both the travelling-wave dynamics and the transverse spectrum; the sign and magnitude of the spectral correction are determined by the finite-γ problem and are not fixed by the incompressible dispersion relation alone.

Several limitations delineate directions for future work. The present calculation is linear and concerns the onset of the transverse instability; nonlinear mode selection, saturation and possible coarsening require longer-time analysis, potentially in three dimensions. Additional biological mechanisms, including phenotypic switching, anisotropy, chemotaxis, active stresses and heterogeneous media, may modify the travelling wave and its stability thresholds. Finally, the projected Newton–Krylov method is designed for robust, positivity–preserving solution of the stiff coupled problem, but no discrete energy-dissipation result is claimed; structure-preserving finite-volume or operator-splitting schemes may be preferable when the symmetric gradient-flow structure is central.

In conclusion, the combination of an explicit transverse stability theory, a positivity–preserving implicit solver and numerical validation near the incompressible limit provides a quantitative framework for studying mechanically driven front instabilities in multi-population growth models. The incipient fingers arise from mobility-dependent pressure transmission across a finite layer, while the external free boundary is attenuated relative to the internal interface.

## Data Availability

The numerical datasets generated during the current study to produce the figures and support the findings of this article are available from the corresponding author upon reasonable request.

## Code Availability

The numerical code, parameter files and scripts used to reproduce the simulations and figures presented in this work are available at Ciarletta et al. (2026b).
